# Magnesium Signaling in Plants

**DOI:** 10.3390/ijms22031159

**Published:** 2021-01-25

**Authors:** Leszek A. Kleczkowski, Abir U. Igamberdiev

**Affiliations:** 1 Department of Plant Physiology, Umeå Plant Science Centre, University of Umeå, 901 87 Umeå, Sweden; 2 Department of Biology, Memorial University of Newfoundland, St. John’s, NL A1B3X9, Canada; igamberdiev@mun.ca

**Keywords:** adenylate energy charge, adenylate kinase, cellular magnesium, free magnesium, nucleoside diphosphate kinase, thermodynamic buffering

## Abstract

Free magnesium (Mg^2+^) is a signal of the adenylate (ATP+ADP+AMP) status in the cells. It results from the equilibrium of adenylate kinase (AK), which uses Mg-chelated and Mg-free adenylates as substrates in both directions of its reaction. The AK-mediated primary control of intracellular [Mg^2+^] is finely interwoven with the operation of membrane-bound adenylate- and Mg^2+^-translocators, which in a given compartment control the supply of free adenylates and Mg^2+^ for the AK-mediated equilibration. As a result, [Mg^2+^] itself varies both between and within the compartments, depending on their energetic status and environmental clues. Other key nucleotide-utilizing/producing enzymes (e.g., nucleoside diphosphate kinase) may also be involved in fine-tuning of the intracellular [Mg^2+^]. Changes in [Mg^2+^] regulate activities of myriads of Mg-utilizing/requiring enzymes, affecting metabolism under both normal and stress conditions, and impacting photosynthetic performance, respiration, phloem loading and other processes. In compartments controlled by AK equilibrium (cytosol, chloroplasts, mitochondria, nucleus), the intracellular [Mg^2+^] can be calculated from total adenylate contents, based on the dependence of the apparent equilibrium constant of AK on [Mg^2+^]. Magnesium signaling, reflecting cellular adenylate status, is likely widespread in all eukaryotic and prokaryotic organisms, due simply to the omnipresent nature of AK and to its involvement in adenylate equilibration.

## 1. Introduction

Magnesium (Mg) is one of the most abundant cations in living cells, second only to potassium [1]. Total Mg concentration in plant cells is in the range of 15–25 mM, and most of it is stored in vacuoles, away from metabolism [2]. A substantial pool of total cellular Mg is required to synthesize chlorophyll in photosynthetic tissues, and the rest is used for ribosome bridging during translation and for chelation with nucleotides, nucleic acids and other phosphate-containing compounds. Normally, as much as 20% of total Mg is in chloroplasts, but it may increase to 50% under low light conditions or during Mg deficiency [3]. Many enzymes require the binding of Mg for activity and/or regulation [4,5]. Processes such as phloem loading [6,7,8], leaf senescence [9], stomata opening and ionic balance of the cell [10,11] are only a few of many examples that illustrate the requirement for adequate Mg homeostasis.

Chelation of nucleotides by Mg is an essential feature of cell metabolism. Among nucleotides, adenylates (ATP, ADP, and AMP) are the most abundant, with ATP being produced both during the reductive (photosynthetic light reactions) and oxidative (respiration) phosphorylation [12]. Most, if not all, enzymes which require ATP in fact use it in its chelated form (MgATP). Assuming that, in leaves, the concentration of free Mg (Mg^2+^) is in the order of 1–5 mM [13], 95–99% of ATP is predicted to be complexed as MgATP. A similar situation occurs with other nucleoside triphosphates (NTPs) [14]. In some compartments (e.g., cytosol) and under specific physiological conditions, the internal [Mg^2+^] may decrease well below 1 mM, e.g., to 0.2 mM in chloroplasts during the induction phase of photosynthesis (see below). Under these conditions, the proportion of Mg-chelated NTPs decreases. The same rule applies to ADP and other nucleoside diphosphates (NDPs), and to a certain extent to AMP and other nucleoside monophosphates (NMPs).

**Figure 1:**
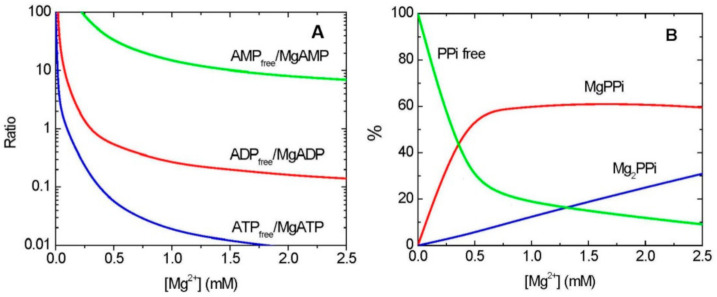
Effects of Mg^2+^ on Mg-chelation with adenylates and PP_i_. (**A**) The ratios of free and Mg-bound adenylates, depending on [Mg^2+^]. (**B**) The percentage of free and Mg-chelated PP_i_, depending on [Mg^2+^]. All lines were drawn according to the values of stability constants for chelation of adenylates and PP_i_ with Mg [15], using Origin software (OriginLab Corporation, Northampton, MA, USA).

In many cases, it is MgADP rather than free ADP which is a substrate/product in enzymatic reactions, whereas AMP binds Mg very weakly, and thus is used in metabolism predominantly as free AMP (Figure 1A). The binding of ATP and Mg is very tight, with stability constant (*K*_MgATP_) of 73 mM^−1^, which implies that a 1:1 (molar ratio) mixture of Mg and ATP will result in nearly all of Mg and ATP tied up as MgATP. The binding of ADP (or other NDPs) by Mg is less strong, with stability constant (*K*_MgADP_) of 4 mM^−1^. This implies that, in physiological conditions, roughly 30% (or less) of ADP exists in the cell as free ADP, and 70% (or more) as a complex with Mg (MgADP) (Figure 1A) [15]. The uncomplexed forms of both ATP and ADP may cause appreciable inhibition of many enzymes, especially the kinase-type phosphotransferases [16]. The binding of Mg to inorganic pyrophosphate (PP_i_), another high energy storing compound, is also highly dependent on [Mg^2+^] (Figure 1B).

Both Mg-complexed and free adenylates are equilibrated by adenylate kinase (AK), a ubiquitous enzyme present in all organisms. The result of AK-maintained equilibrium, [Mg^2+^], is set as a signal from total adenylate pool in a given cellular compartment. The [Mg^2+^], in turn, depends on metabolic status of the cell, and on rates of transport of Mg^2+^ and adenylates on specific transporters (see below). AK in different cell compartments establishes the concentration of [Mg^2+^] via equilibration of adenylate species, as well as membrane potential of cell organelles [17], and the equilibrium of AK determines [Mg^2+^] in a controlled way depending on the rates of ATP production and consumption and, in turn, optimizing these rates. This [Mg^2+^] directly regulates multiple enzymes and translocators, thus representing a powerful feedback signal from the energy level of the cell and its compartments expressed in the concentrations of adenylate species.

Mg^2+^ has previously been suggested as a signal in human T cell immunodeficiency, with the plasma membrane-bound Mg^2+^ transporter identified as a major player [18]. We argue here that the Mg^2+^ signaling is in fact widespread in plants and, probably, in other organisms as well, and that it is a simple consequence of the status of Mg-free and Mg-complexed adenylates in a given cell or tissue, which is maintained via AK. AK has previously been implicated as a central hub in the concept of “adenylate charge” theory [19], but also as a regulator of AMP and ATP signaling in plants and animals [20,21].

In this review/opinion paper, we have focused on magnesium and cell energetics in plants, but we also refer to other organisms, when applicable.

## 2. AK and NDPK in Cell Energetics

In plant cells, AK activity is widely distributed through different compartments, namely plastids, mitochondria, nuclei, and cytosol [22,23,24,25,26]. Different isozymes of leaf AK were purified from various species using affinity chromatography [27]. Arabidopsis contains a total of eight genes of AK. Of those, at least two genes code for plastidial AK isozymes [23,25], at least three genes code for mitochondrial AKs [25,28], and one gene codes for nuclear AK [26]. The remaining two genes code for AKs which may be dually targeted between mitochondria and plastids, and mitochondria and cytosol, respectively [25] (Table 1). Mitochondrial AKs are believed to be located in the space between outer and inner membranes (the intermembrane space; IMS), possibly membrane-bound, but not in the mitochondrial matrix [25]. The pool of free and Mg-bound adenylates in the matrix, despite the lack of matrix-own AK, is affected by AKs from mitochondrial IMS via the involvement of adenylate translocators in the inner mitochondrial membrane [12,14]. Contrary to plants, human mitochondria do contain an AK isozyme in the matrix [29].

Plant AKs react almost exclusively with AMP and ATP as substrates [22,26,32]. Human AKs, however, while more or less specific for AMP, can react with a variety of NTPs, depending on the AK isozyme. Thus, erythrocyte and serum AKs react only with ATP; muscle AK preferentially reacts with ATP and, to some extent, other NTPs; and liver AK reacts with UTP and GTP [33].

The roles of plant AKs, as determined using knockout mutants, are listed in Table 1. Whereas the disturbance of adenylate equilibrium in a given compartment in such mutants and the resulting effects on cell energetics are most likely the major causes of a given phenotype, a role of AK protein itself as a regulator cannot be discounted. One example for this is the reported complexation of AK with chloroplast glyceraldehyde-3-phosphate dehydrogenase, with the complex proposed to optimize photosynthesis during rapid fluctuation in environmental resources [34]. Another example concerns a plant nucleus-associated AK6 isozyme, which is homologous to human nuclear AK6 [26,31]. The plant protein has AK activity and was found to be essential for stem growth in Arabidopsis [26], but also contributing to Arabidopsis root growth control [31]. Plants lacking AK6 over-accumulated 80S ribosomes relative to polysome levels, consistent with the AK6 role in ribosome maturation [31]. In yeast, an orthologue of AK6 encodes the well-characterized ribosome assembly factor Fap7 [35], which has been reported to mediate cleavage of 20S pre-rRNA by directly interacting with an Rps protein. Using pulldown and two-hybrid system, the Arabidopsis AK6 was found to interact physically with Arabidopsis’ own Rps14 [26]. Interestingly, it has been earlier observed that different plant AK isozymes can bind RNA and that, at least in plants, RNA-binding by AK may be related to regulatory mechanisms [36].

Aside from equilibrating adenylates, in some tissues AK was also reported to act more unidirectionally toward either ATP formation or utilization. For instance, under some conditions, e.g., in drying seeds where tissue dehydration leads to a decline in mitochondrial energy production, AK may become the main ATP (and AMP) producing mechanism [37]. Following imbibition, however, cellular adenylate balance is rapidly restored from AMP by both AK and oxidative phosphorylation in mitochondria. Another study involved antisense inhibition of the expression of plastidial AK in potato tubers, which led to dramatic effects on the overall metabolism and tuber yield [38]. In field trials, the transgenic plants had up to a 10-fold increase in ADP-glucose (key precursor to starch synthesis [39]), a 2–4-fold increase in some amino acids, and an almost two-fold increase in starch, with tuber yield nearly doubled when compared to WT plants. This suggested that, under in vivo conditions, the amyloplastic AK acts in the ATP-consuming direction, and competes for ATP both with ADP-glucose pyrophosphorylase, which produces ADP-glucose, and with plastidial pathways of amino acid biosynthesis [38].

Whereas ATP is the only NTP which arises via photophosphorylation (in chloroplasts) and oxidative phosphorylation (in mitochondria) [40], other NTPs in plants are formed mainly via nucleoside diphosphate kinase (NDPK) activity [14,41,42]. Its reaction can be described as: ATP + NDP ↔ ADP + NTP. In animals and bacteria, AK can apparently substitute for NDPK, due to the apparent bifunctionality of AKs in those organisms [43]. In plants, however, where AK is specific for adenylates, these two activities are frequently metabolically “coupled” together [42,44,45], which may involve physical interaction [46]. Both AK and NDPK have been linked to stress perception [47], and they are major components of the so-called cell thermodynamical buffering system [14,48,49], which has been proposed to operate during photosynthesis and respiration [12,50,51], and, arguably, during starch synthesis in plastids and during cell wall polysaccharide formation in the plasma membrane and endoplasmic reticulum (ER) [42].

Both AK and NDPK are functionally coupled to photophosphorylation and oxidative phosphorylation, establishing that concentrations of nucleoside phosphates depend on the rates of ATP synthesis and consumption, and optimizing the operation of ATP synthases [12]. Plants contain several genes for NDPK, e.g., five genes in Arabidopsis and rice, coding for different isozymes located in cytosol, plastids, mitochondria and, possibly, ER [41]. In potato roots, cytosolic NDPK activity is believed to supply UTP for the reaction of UDP-glucose pyrophosphorylase (UGPase), to produce UDP-glucose, a key precursor to sucrose and cell wall polysaccharides [52]. Additionally, in cereal seeds, cytosolic NDPK may provide UTP which, indirectly, is used for starch synthesis [44].

## 3. AK-Mediated Adenylate Equilibrium and Mg^2+^ Signaling

**Figure 2:**
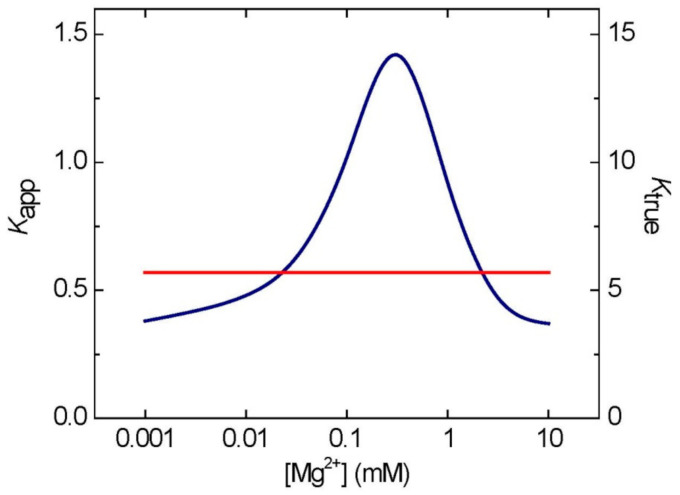
Effects of [Mg^2+^] on *K*_app_ and *K*_true_ of AK. The *K*_app_ peaks at ca. 0.2 mM Mg^2+^. Please note that the scale on X-axis is logarithmic. The lines for *K*_app_ (blue) and *K*_true_ (red) were computed as described in ref 55, using Origin software (OriginLab Corporation, Northampton, MA, USA).

The AK reaction has frequently been presented as: 2 ADP ↔ ATP + AMP [19]. However, the true substrates/products of AK are: MgADP + ADP ↔ MgATP + AMP [53,54]. An important consequence of this is that apparent equilibrium constant (*K*_app_) of the first reaction (with total adenylates), where *K*_app_ = [ATP][AMP]/[ADP]^2^, depends on free magnesium (Mg^2+^) concentration in the reaction mixture and can be described as a bell-shaped curve, peaking at 1.5 at ca. 0.2 mM Mg^2+^ (Figure 2). At any other value of [Mg^2+^], the *K*_app_ corresponds to two values of [Mg^2+^], one below and one above 0.2 mM. In studies with plant material, the right side of the bell-shaped curve is most relevant, because internal [Mg^2+^] in plants is usually above 0.2 mM [55]. The AK equilibrium-linked [Mg^2+^] values can be easily computed from the following Equation (1)

[Mg^2+^] = [0.7 − 0.25*K*_app_ ± 0.57(1.5 − *K*_app_)^1/2^]/(*K*_app_ − 0.1) (1)

where a given *K*_app_ of AK can be calculated from the experimentally determined total of each of the adenylates taking part in the reaction (AMP, ADP, and ATP) [55]. In contrast to its *K*_app_, the true equilibrium constant of AK, defined as *K*_true_ = [MgATP][AMP]/[MgADP][ADP], is not dependent on [Mg^2+^] and has a fixed value of ca. 5.5 (Figure 2). The non-linear relationship between *K*_app_ and [Mg^2+^] was observed in several studies with purified AKs [16,53,54,56,57], and similar principles most likely also apply under in vivo conditions [14,49].

As evident from Equation (1), the *K*_app_ of AK, although different from the *K*_true_, can be a useful parameter linking concentrations of all adenylates to [Mg^2+^]. Thus, knowing the contents of total ATP, ADP, and AMP in a given biological preparation, and assuming that they are under equilibrium governed by AK, can provide information about intracellular [Mg^2+^]. Similarly, when only [Mg^2+^] is known, this can be linked with a given *K*_app_ of AK (Figure 2). A similar computational set can be established for Mg^2+^ and other nucleotides (guanylates, cytidylates, and uridylates) via the corresponding buffering equilibria of NDPK and UMP/CMP kinase. The latter reacts reversibly with UMP and CMP rather than AMP, and uses ATP as a second substrate [25,58]. It is unknown whether the UMP/CMP kinase reaction requires a combination of Mg-chelated and free nucleotides, as is the case for AK, but magnesium is apparently required for the reaction [58].

When considering the Mg requirement for NDPK, its true reaction can be described either as MgATP + NDP ↔ ADP + MgNTP, or MgATP + MgNDP ↔ MgADP + MgNTP. To the best of our knowledge, it is unknown whether nucleoside diphosphates (including ADP) used by NDPK are reactive as Mg-chelated or Mg-free species. This could, at least theoretically, make significant difference in terms of the relationship between the *K*_app_ of NDPK and [Mg^2+^]. With respect to UMP/CMP kinase, the *K*_app_ of its reaction (UMP/CMP + ATP ↔ UDP/CDP + ADP) is likely to depend on [Mg^2+^] in the same way as AK. Additionally, regardless of what true substrates are for NDPK and the other kinase, cellular pools of non-adenylate nucleotides are generally much smaller than those of adenylates [42]. Thus, intracellular [Mg^2+^] would still respond more strongly to AK-mediated equilibrium than that of NDPK and UMP/CMK kinase.

## 4. Magnesium and the Adenylate Energy Charge Theory

Equilibrium of adenylates maintained by AK is at the core of the adenylate energy charge (AEC) theory, developed and popularized by Atkinson [19]. The theory assumes that AK uses total adenylates as substrates and that the adenylate concentrations at AK equilibrium account for the energy status in metabolism. This can be defined as AEC = ([ATP] + ½ [ADP])/([ATP] + [ADP] + [AMP]), with the concentrations of adenylates equilibrated by AK reaction. The theory has been criticized [16,59,60] on the grounds that it does not take into account a crucial role of magnesium for the AK reaction. Most importantly, mass action (*K*_app_) of AK is very much dependent on [Mg^2+^] (Figure 2), and is not constant, as assumed for AEC. As a consequence of that, at low [Mg^2+^], as it is in the cytosol, free ATP may actually inhibit MgATP-utilizing enzymes rather than serving as a substrate. The same concerns glycolytic kinases involved in ATP formation, which use MgADP rather than free ADP as a substrate, with [Mg^2+^] being a key player in controlling MgADP availability [53]. These and other arguments against AEC as a key parameter controlling energy status of cellular processes have been summarized by Purich and Fromm [16,59] and Pradet and Raymond [60], and we will not cover them here. However, it is important, in our opinion, to emphasize that Mg signaling as controlled by AK is not compatible with AEC theory. This is simply because the AEC does not take into account true substrates (Mg-bound and Mg-free) of AK and the crucial role of [Mg^2+^] in making these substrates available for AK.

## 5. Magnesium Status in Cells

With a total cellular concentration of magnesium at 15–25 mM with 15–20% bound to chlorophyll [61] and free magnesium concentration frequently at a less than millimolar level [62,63], this implies that most of magnesium is complexed, and only a small fraction exists as Mg^2+^. It has been reported that up to 90% of the cytosolic pool of nucleotides is bound to Mg [64]. The same applies to chloroplasts [13], and probably to all other compartments which contain metabolically active nucleotides. For instance, in chloroplast stroma, free Mg^2+^ ranges from ca. 0.2 to 5 mM (Table 2), with the lower values characteristic for darkened leaves, and the higher values for illuminated leaves [55,65,66]. This constitutes less than 10% of total Mg in chloroplasts. The rest is confined mostly to chlorophyll in thylakoids, but also chelates stromal pools of phosphorylated compounds (e.g., ATP) and dicarboxylic acids [65].

A major role in Mg^2+^ homeostasis in plants belongs to the vacuole [11]. Vacuoles buffer and balance fluctuating concentrations of external nutrients, but they can also alleviate the effects of excessive concentrations of such compounds. In Arabidopsis leaves fed with high-Mg–sap solutions, vacuoles may accumulate up to 80 mM Mg [69]. This concentration is one order of magnitude higher than vacuolar [Mg] under normal conditions [2]. Upon withdrawal of Mg from the nutrient solution, a typical first symptom of Mg deficiency is the higher accumulation of starch and sucrose in the leaves, followed by leaf chlorosis, which may lead to a lower photosynthetic rate [1]. It has been suggested that Mg deficiency limits the carbohydrate transport from source organs to the sink by affecting the loading of sucrose to the phloem, which requires an adequate Mg concentration [6,7,8]. It has been reported that the process of Mg translocation can be hampered under severe Mg-deficiency, having effects both on photoassimilate partitioning and root growth [70]. The disruption of Mg transport in young plants could result in reduced growth of the plant at a later growth stage.

Cells are usually quite resistant to external Mg-deficient conditions. As pointed out by Gout et al. [67], it takes 14 days to decrease cellular magnesium content by five-fold, when placing sycamore cells into Mg-free media. They also found that during first 10 days of Mg-deficient conditions, the cytosolic [Mg^2+^] did not change at all due to a release of Mg^2+^ from the vacuole. Only after 10 days was there a decrease in cytosolic [Mg^2+^], accompanied by a cessation of cell growth and a decrease in respiration.

To the best of our knowledge, there are no data on [Mg^2+^] in the nucleus, ER lumen, and peroxisomes. For the nucleus, we can only assume that its [Mg^2+^] is similar to that in the cytosol, given the porous structure of the nuclear envelope. In addition, the nucleus contains its own AK [26], which probably can access the same adenylate pool (given the pores in nuclear membrane) as in the cytosol. For the ER, the major obstacle for the determination of Mg^2+^ has been the presence of high (millimolar) concentration of Ca^2+^, preventing reliable Mg^2+^ detection there with the use of ionophores [71]. However, the ER has at least one Mg^2+^ transporter (see below), and metabolism within ER strongly depends on ATP supply [72,73], suggesting an important role for Mg^2+^ in this organelle. Peroxisomes probably have low [Mg^2+^], because of the lack of an Mg^2+^ transporter in their membrane, but they also constitute only a tiny fraction of cell volume, thus are unlikely to contribute significantly to overall cellular Mg^2+^ homeostasis. Both ER and peroxisomes do not have their own AK, and adenylate metabolism there must be independent of AK equilibrium.

## 6. Feasibility of the Estimations of [Mg^2+^] Based on Adenylate Measurements

Assays of intracellular [Mg^2+^] in plants most frequently have been performed with Mg-binding ionophores, using fluorescence spectrophotometry to detect the Mg–ionophore complex, sometimes also with help of fluorescence microscopy [66,74,75]. However, many of the Mg^2+^ fluorescent probes proved unsatisfactory, due to their lack of specificity or low affinity for Mg^2+^ [74]. Additionally, the ionophores need to be loaded into cells before the assays, which may perturb normal metabolism. The use of ^31^P-NMR was more successful, and it permitted non-invasive in vivo studies, allowing simultaneous identification and quantification of free and Mg-complexed nucleotides as well as [Mg^2+^] in whole cells, the cytosol, and organelles [63,76]. Those and other methods of Mg determination in biological samples, e.g., electron probe X-ray microanalysis (XRMA) or ^13^C-NMR citrate/isocitrate ratio, have been discussed by Romani and Scarpa [77], and they all require specialized scientific tools and expertise. On the other hand, adenylates can be easily quantified by a variety of methods, using standard laboratory equipment. Although an indirect measure, the calculated [Mg^2+^] values that were derived from adenylate contents are comparable to those obtained by other methods (Table 2).

Scientific literature abounds with measurements of adenylate species in whole organs/ tissues and, to a lesser extent, in fractionated preparations containing purified organelles [55,78]. These data can be recalculated for estimations of [Mg^2+^], especially in organelles. Based on its subcellular localization, the AK-mediated equilibrium of adenylates and the resulting Mg^2+^-signaling encompasses chloroplasts (both stroma and IMS), cytosol, nucleus and the IMS of mitochondria (Table 1)). The outer membranes of chloroplasts and mitochondria are permeable to small compounds, e.g., adenylates, and thus the IMS in both organelles is under AK equilibrium, which extends through the permeable outer membranes to the cytosol [62,67]. Adenylate data collected for any of these compartments should tightly correlate with an internal [Mg^2+^] there; as indeed is the case when compared to other methods of [Mg^2+^] determination (Table 2).

A different situation occurs if the adenylate data are collected for whole tissues, cells, or protoplasts, where mixing up of various adenylate pools occurs, and the final result reflects the adenylate status in whole tissue/cells, but not in given compartments. The calculated *K*_app_ of AK for such a system will be a mean *K*_app_ of all cellular AKs and should apply only to compartments where AK equilibrium is established. This excludes peroxisomes, ER, and vacuoles, which lack AK isozymes (Table 1). Among those organelles, vacuoles do not have any adenylate translocators and, besides, it has already been reported that potato tuber vacuoles contain no adenylates [79]. However, both peroxisomes and ER do have adenylate translocators (see below), and thus adenylates present in these organelles may affect the *K*_app_ of AK based on total contents of adenylates from whole plant cells/tissues. The contribution of peroxisomal adenylates is probably close to negligible (small size of peroxisomes), whereas the pool of adenylates in the ER might be significant, given that the ER represents a continuous membrane system, often quite abundant in the cytosol.

Thus, in summary, when using the concept of the *K*_app_ of AK to derive [Mg^2+^] from total contents of adenylates in tissues or whole cells, the main drawback of this method is that it yields an average [Mg^2+^] for all compartments where AK equilibrium applies (thus excluding vacuoles, ER, and peroxisomes). This approach is obviously only an approximation (e.g., possible error from the contribution of ER’s adenylates), but it could be useful, especially when studying a process known to be confined to a single compartment, where substantial changes in [Mg^2+^] have already been observed by other methods, e.g., during the light-induction phase of CO_2_ fixation in chloroplasts [55] or during anoxia in the cytosol [50].

In earlier studies [55], based on published data for adenylate contents in plant tissues and organs, we have found that levels of Mg^2+^ in leaves depend on the developmental stage (young leaves having lower [Mg^2+^] than old ones), light conditions (darkened leaves have lower [Mg^2+^] than in illuminated ones), and salt stress conditions (stressed plants have lower [Mg^2+^]). For instance, based on data from Nieman et al. [80], [Mg^2+^] in young and mature pepper leaves was 3 and 6 mM, respectively, whereas salt stress decreased [Mg^2+^] in safflower buds (from 10 to 4 mM). When applying the same approach to adenylate data from one study on the effects of salt (NaCl) stress on energetics of cyanobacteria [81], the calculations again suggest strong effects of salt on internal [Mg^2+^]. When stressed, the cyanobacteria maintained low [Mg^2+^] levels of 0.24 mM, which markedly increased to 2.2 mM upon salt withdrawal. This probably reflects the observed two-fold higher photosynthesis rate of these microorganisms under normal conditions, resulting in more ATP produced and thus requiring more magnesium. Cyanobacteria are prokaryotes and do not have any organelles; therefore, aside from thylakoid-like membrane system, the calculated [Mg^2+^] might be representative of [Mg^2+^] anywhere inside of their cells.

## 7. Magnesium Translocators

**Figure 3:**
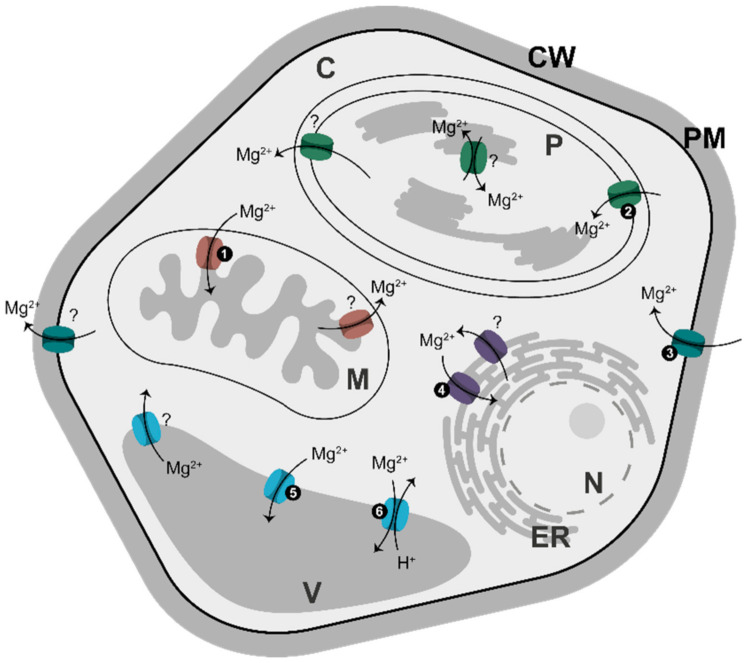
Distribution of Mg^2+^ transporters in membranes in plants. All transporters shown here have been identified in Arabidopsis [83]. The light-grey area corresponds to compartments where AK equilibrium is established. Numbers correspond to: (1) MGT5; (2) MGT10; (3) MGT1, MGT5, MGT6, MGT9; (4) MGT4, MGT7; (5) MGT2, MGT3; and (6) MHX. Question marks refer to transporters involved in Mg^2+^ export from a given compartment/cell; their nature remains unclear. Abbreviations: C, cytosol; CW, cell wall; ER, endoplasmic reticulum; M, mitochondrion; N, nucleus; P, plastid; PM, plasma membrane; V, vacuole.

Soils are usually low in Mg content, because Mg binds soil only weakly and can be easily leached out by rains. To adapt to such conditions, plants have evolved a highly efficient system for Mg acquisition from the soil; its transport via xylem; and its distribution to targeted tissues/cells. This system has been comprehensively reviewed [2,4,82], and we will not cover it in this paper. Instead, we will focus on Mg^2+^ traffic into and within a cell. Mg^2+^ is taken up first by specific translocators in the plasma membrane, and then it is distributed to several membrane-surrounded compartments, each using its own specific set of Mg^2+^ transporters (Figure 3).

Most Mg^2+^ transporters belong to a single family of proteins, which in turn belongs to the CorA protein superfamily [84]. In plants, this family was first described in Arabidopsis by two groups, which named it *At*MRS2 [85] and *At*MGT [84]. For simplicity, we will refer to those transporters as belonging to the MGT family. In Arabidopsis, there are 10 genes for MGT [86], whereas in rice nine genes have been identified [87]. Most of the Mg^2+^ transporters are responsible for Mg^2+^ import into a given compartment, and the rest are involved in Mg^2+^ export. The importers are relatively well described, whereas the nature of Mg^2+^ exporters is less clear [83]. Some of the importers may become exporters, depending on [Mg^2+^], as is the case for *At*MGT5, which has a dual role as an Mg-importer at micromolar levels, and an exporter at a millimolar range [86]. Besides Mg^2+^, some MGT members may also transport other cations, including Zn^2+^ and Cu^2+^. There are also other carriers predominantly transporting K^+^ or Ca^2+^, but which are also permeable to Mg^2+^. Non-selective cation channels are the other candidates for Mg^2+^ transport [1,88]. In addition, Mg^2+^ availability may affect the activities of plasma membrane transporters for Ca^2+^, K^+^, and H^+^ [88].

Under conditions of Mg excess, as in so called serpentine soils [89], plants deploy an elaborate system to avoid Mg toxicity. This system is composed of plasma membrane- and tonoplast-localized calcineurin B-like proteins (CBLs) and their downstream components, CBL-interacting protein kinases (CIPKs) [90]. At high external Mg^2+^, there is an interaction between the plasma membrane-associated CBL and CIPK components which modulates the activity of a number of ion channels/transporters, facilitating the uptake or exclusion of Mg^2+^. In the next step, high cytosolic [Mg^2+^] triggers changes in internal Ca^2+^, which are then sensed by tonoplast CBLs. This, in turn, triggers tonoplast CIPKs to activate Mg^2+^ transporters or channels to detoxify the cytosol from Mg^2+^ [90]. The CBL/CIPK system may also have a similar role in protecting against the toxicity of other ions, including excess of Na^+^ ([90,91,92,93]. Some studies, based on knockout mutants, have identified plasma membrane-bound MGT6 and ER-associated MGT7 as likely candidates involved in the detoxification of Mg^2+^ [93]. MGT6 was also required for plant adaptation to a low [Mg^2+^] [94].

Arabidopsis contains four MGT proteins in the plasma membrane, whereas vacuolar tonoplast contains two MGT transporters (MGT2 and MGT3) [69] and the so-called MHX transporter [95] (Figure 3). MHX is structurally distinct from MGT/MRS2 transporters and shows the highest similarity to mammalian Na^+^/Ca^2+^ exchangers, which are part of the Ca^2+^/cation (CaCA) exchanger superfamily [96]. The MHX protein exchanges vacuolar protons for cytosolic Mg^2+^ and Zn^2+^ [95,97]. In Arabidopsis, MHX co-localizes with a major chromosomal quantitative trait locus (QTL), affecting seed Mg content [98].

Besides being located in the plasma membrane and tonoplast, Mg^2+^ transporters are also elsewhere in the cell, i.e., in plastids, mitochondria and ER (Figure 3). Some of the transporters are tissue-specific, e.g., Arabidopsis mitochondrial MGT5 which is exclusively expressed in anthers at early stages of flower development, underlying its role in pollen development and male fertility [86]. MGT4 in the ER and MGT9 in the plasma membrane are also essential for pollen development [82,83].

Exact subcellular location needs to be reevaluated for some Mg^2+^ translocators, because several studies have reported discrepant results, especially concerning putative ER-location. As pointed out by Yan et al. [93], membrane proteins can be mis-targeted to ER, especially when overexpressed in a transient expression system. It would also be interesting to see how plants that have adapted to growth on serpentine soils and deal with excess Mg^2+^ are managing their AK-mediated energy metabolism.

## 8. Adenylate Translocators

**Figure 4:**
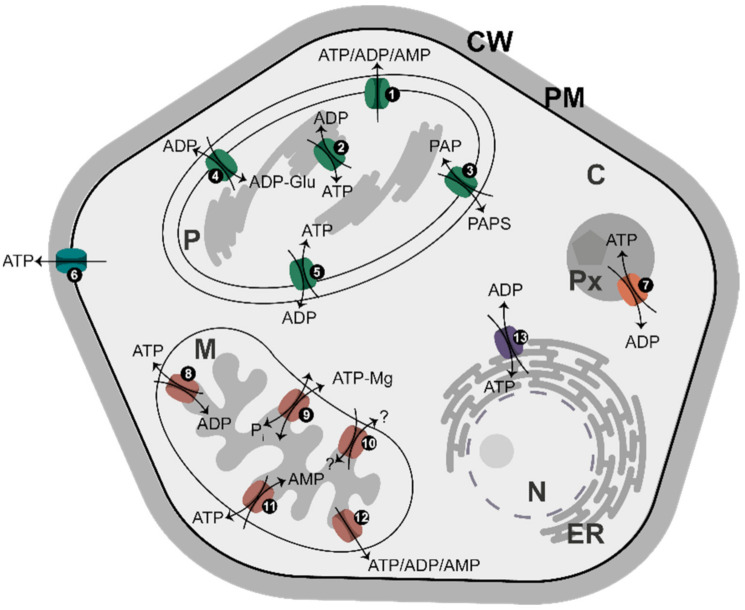
Distribution of major adenylate carriers in membranes in plants. The names of the transporters and major transported molecules are as they are given by da Fonseca-Pereira et al. [99]. The light-grey area corresponds to compartments where AK equilibrium is established. Numbers correspond to: (1) AtBT1*, Arabidopsis thaliana* ATP/ADP/AMP carrier; (2) ATP/ADP carrier; (3) TAAC/PAPST1; (4) ZmBT1, maize (*Zea mays*) plastid ADP-Glucose/ADP carrier; (5) NTT1-2, ATP/ADP carriers; (6) PM-ANT1; (7) PNC1-2, ATP/ADP carrier; (8) AAC1-3, ATP/ADP carriers; (9) APC1-3, MgATP/P_i_ carriers; (10) ZmBT1, maize mitochondrial transporter, the substrate and transport mode of which are unclear; (11) ADNT1, AMP/ATP carrier; (12) AtBT1, ATP/ADP/AMP carrier; and (13) ER-ANT1, ATP/ADP carrier. Abbreviations: C, cytosol; CW, cell wall; ER, endoplasmic reticulum; M, mitochondrion; N, nucleus; P, plastid; PAP, 3’-phosphoadenosine 5’-phosphate; PAPS, 3’-phosphoadenosine 5’-phosphosulfate; PM, plasma membrane; Px, peroxisome.

In Arabidopsis, at least 16 distinct genes for adenylate carriers have been identified [99], which code for proteins distributed in the plasma membrane, plastids, mitochondria, peroxisomes, and ER (Figure 4). Together, they represent an efficient system of energy partitioning between different cell compartments. Most of them are antiporters, transferring one adenylate in exchange for another adenylate species (or inorganic phosphate, P_i_, as is the case for some mitochondrial carriers). In most cases, ATP is exchanged for ADP, but there are also uniporters for ATP transport (at the plasma membrane) or for the transport of all adenylates (ATP, ADP, and AMP) in plastids and mitochondria. Importantly, mitochondria have an antiporter transporting ATP in exchange for AMP and, to some extent, ADP [99]. It is believed that adenylate translocators, with the exception of MgATP/P_i_ exchangers [99], use free adenylates for transport across a given membrane [62,100], implying a major role for [Mg^2+^] in regulating a supply of free adenylates to the translocators.

Adenylate translocators have variable organ/tissue-specific expression patterns under different environmental conditions and at different developmental stages, suggesting specific non-redundant functions for each of the translocators [99]. For instance, the chloroplast ATP/ADP antiporter has been identified as one of several membrane-bound proteins exhibiting increased abundance after cold acclimation [101]. In earlier studies on AK, a possible link was found between activities of certain AK isozymes and adenylate transport during plant flowering [102]. Upon flower induction, although total AK activity in the leaves and stems remained the same, the intracellular distribution of AK activity changed, with the most prominent being a strong decrease in activity of one of chloroplast AK isozymes. This AK was proposed to functionally interact with the chloroplast adenylate translocator, responding to alterations in energy distribution between chloroplast and cytosol during floral induction [23,102].

## 9. Role of [Mg^2+^] in Metabolism and Signaling

There are several aspects to the involvement of Mg in cell energetics: (i) Cellular ATP (and to some extent ADP) is strongly chelated by Mg^2+^, and the chelated and free nucleotides are frequently key substrates/effectors in metabolism. The same concerns pyrophosphate (PP_i_), an alternative energy currency, which is active as an Mg-chelated or Mg-free species; (ii) Binding of Mg^2+^ frequently modulates and stabilizes activities of enzymatic proteins involved in cell energetics processes, but also in DNA replication, transcription and translation and other processes; (iii) The Mg-chelated and free adenylates govern various aspects of cell energetics, such as rates of energy metabolism, translocation of adenylates across membranes, or contribute to allosteric regulation of metabolism; (iv) The substantial changes in intracellular [Mg^2+^], as in the cytosol of cells under anoxia, may reflect switches in metabolism between MgATP-based and MgPP_i_-dependent; and (v) Mg^2+^ affects concentrations of Ca^2+^ and other cations, and alleviates the effects of stress by excess [Na^+^]. Below, we will briefly describe each of these aspects.

### 9.1. Mg^2+^ and Chelation of Adenylates and PP_i_

Changes in subcellular [Mg^2+^] have significance in establishing the approximate distribution of ATP and ADP among the Mg-free and Mg-complexed forms. For instance, certain kinases, e.g., pyruvate kinase or phosphoglycerate kinase, that use ADP to produce ATP in the process of substrate phosphorylation, react with MgADP rather than ADP as their substrate [53,103]. Under low [Mg^2+^], these reactions will be limited because of shortages of MgADP and an excess of free ADP, which likely acts as inhibitor. On the other hand, the MgADP complex will be sensitive to changes in concentrations of Mg^2+^ and total ADP, both of which change reciprocally with changes in total ATP. In some instances, MgADP can act as an inhibitor, as is the case for several MgATP-utilizing enzymes in the cytosol which are competitively inhibited by MgADP [63,104]. For these enzymes to operate effectively, it is very important that cytosolic [Mg^2+^] is maintained at low levels, which implies that MgADP will also be low. Under hypoxia or anoxia conditions, however, cytosolic [Mg^2+^] markedly increases, which leads to increases in [MgADP], which may affect hexokinase activity, and thus glycolysis [105]. Additionally, changes in cytosolic [Mg^2+^] may impact protein kinase activities and subsequent signal transduction. Mg^2+^ plays an important role in the ATP binding in the active site of the kinase and facilitates phosphoryl transfer reactions [106].

PP_i_, an alternative (to ATP) energy currency, is produced mainly during fatty acid and amino acid activation for the degradation of fatty acids and for protein synthesis, respectively, and during nucleic acids synthesis [107]. Important additional sources of PP_i_ are various pyrophosphorylases which, in addition to PP_i_, produce a variety of nucleotide sugars (e.g., UDP-glucose). In all these reactions, a subsequent hydrolysis of PP_i_ into two P_i_ molecules by a pyrophosphatase (PPase) [108,109] or PP_i_ removal by other PP_i_-utilizing enzymes [44] drives the overall metabolism toward the activated substrate formation.

PP_i_ can be used as an energy source instead of ATP, when the latter supply is low and when cytosolic [Mg^2+^] increases, as in anoxia/hypoxia. In such cases, PP_i_ is frequently used as a substrate, as MgPP_i_, rather than free PP_i_ (Figure 1B), as in the reaction of PP_i_-dependent phosphofructokinase [110]; free PP_i_ acts as the inhibitor of this enzyme [111]. The vacuolar H^+^-PPase uses Mg_2_PP_i_ (Figure 1B) as its substrate [112,113] and is allosterically activated by Mg^2+^ [114]. This means that the PPase needs an increased [Mg^2+^] for its optimal operation, providing a link to the hypoxic metabolism characterized by Mg^2+^ release upon the decrease in ATP production [50]. Another example of the different requirements for Mg^2+^ is provided for several aminoacyl-tRNA synthetases, key activities producing direct precursors for protein synthesis. In their reverse reaction (pyrophosphorolysis), one group of aminoacyl-tRNA synthetases uses MgPP_i_ and the other prefers Mg_2_PP_i_ [115], implying that they will be fully active only at specific (and different) Mg^2+^ concentrations. MgPP_i_ serves also as a substrate for non-proton pumping PPases [116,117] and several other PP_i_-utilizing enzymes [42,50,109].

Stability constant for formation of MgPP_i_ is much lower than that for MgATP (*K*_MgPPi_ of 1.2 mM^−1^ vs. *K*_MgATP_ of 73 mM^−1^) [15]; therefore, this implies that MgPP_i_-utilizing enzymes will operate effectively only at a relatively high [Mg^2+^], and even small changes in intracellular [Mg^2+^] (below ca. 0.7 mM) may have significant effect on the MgPP_i_ availability (Figure 1B). Excess of PP_i_, however, can be lethal, disrupting metabolic pathways. In Arabidopsis plants impaired in cytosolic pyrophosphatase, the accumulated PP_i_ inhibited UDP-glucose formation by UGPase [118]. It is unknown whether it is free or Mg-chelated PP_i_ acting as the UGPase inhibitor, but the free PP_i_ appears to be a better candidate, given the low [Mg^2+^] in the cytosol. UDP-glucose is a key direct or indirect precursor to myriads of glycosylation reactions, including the formation of sucrose, starch, but also cellulose, hemicellulose, glycoproteins, and many other carbohydrate-containing end-products [119,120]. All these pathways may, thus, be affected by fine changes in cytosolic [Mg^2+^].

### 9.2. Mg^2+^ as a Regulator of Enzymatic Activities

Earlier, we have identified several reactions involved in carbohydrate synthesis that require magnesium either via complexation with NTP to form MgNTP, a true substrate, or as an effector of a given enzyme (i.e., stimulating or inhibiting a given activity). These reactions included, among others, several NDP-sugar producing pyrophosphorylases and sucrose synthase (SuSy) (for details see ref [42]). The pyrophosphorylases produce nucleotide sugars, which are substrates for glycosylation reactions, whereas SuSy is involved in the metabolism of sucrose, a soluble sugar [120]. Mg^2+^ activates SuSy toward UDP-Glc production and it inhibits the reverse reaction (sucrose formation) [121]. Stimulation by Mg^2+^ was also found for the activity of phosphorylated (soluble), but not for non-phosphorylated (membrane-bound) SuSy [122], suggesting that Mg^2+^ affects sucrose breakdown (soluble SuSy), but not cellulose synthesis (membrane-bound SuSy).

The apparent control exerted by Mg^2+^ over carbohydrate metabolism strongly suggests a dual role of AK and NDPK in this process. Firstly, AK and NDPK produce nucleoside triphosphates as substrates for the pyrophosphorylase reactions; this happens either directly (production of MgATP by AK) or indirectly (production of MgUTP, MgGTP and MgCTP) by linking AK activity, via NDPK, with kinases of uridylate, guanylate, and cytidylate metabolism [42]. The second role of AK (and possibly of NDPK) is its control of [Mg^2+^], which acts as a substrate (complexed with NTP) for the pyrophosphorylases and as an effector for both pyrophosphorylases and SuSy [42].

Among many other examples of control exerted by magnesium [17,49,50,51,55,62], an important case is the functioning of ribozymes [123], in particular in the process of protein synthesis on ribosomes. Most of the studies in this area have been performed in prokaryotes. As we mentioned earlier, aminoacyl-tRNA synthetases belong to two groups with different requirements for Mg^2+^ [115]. In fact, four types of dependencies on Mg^2+^ were observed in these two groups. The class I synthetases require only one Mg^2+^ for the activation reaction (in MgATP), while the class II synthetases require three Mg^2+^ ions (one in MgATP and two in Mg_2_PP_i_). In class II synthetases, both MgPP_i_ and Mg_2_PP_i_ participate in the pyrophosphorolysis of the aminoacyl adenylate, but some of them show a better fit if Mg_2_PP_i_ reacts and others when only MgPP_i_ but not Mg_2_PP_i_ is used in the pyrophosphorolysis. The data for eukaryotic and, in particular, plant enzymes participating in protein synthesis are quite limited; however, the key role of Mg^2+^ has been shown during splicing for the functioning of spliceosome [124].

Aside from the examples presented above, magnesium is also essential for DNA replication and for transcription. Most of the enzymes involved in these processes require Mg either chelated to NTP (which then acts as substrate) or acting as an effector. Crystal structures of DNA polymerases, involved both in replicating DNA and in DNA repair, revealed a crucial role of Mg ions in faithfully positioning a given nucleotide in the active site of the enzyme and promoting phosphoryl transfer [125]. A similar role for Mg was found for RNA polymerase [126]. Biologically active structures of both DNA and RNA are stabilized by Mg [127].

### 9.3. Mg^2+^ Regulates Energy Metabolism, Adenylate Transport, and Allosteric Regulation

Earlier, we summarized effects of different ratios of adenylate species, both free and Mg-complexed, on metabolism, along with formulas for the calculation of adenylate ratios upon AK equilibrium [62]. Thus, the MgATP/MgADP ratio reflects anabolism-driving potential; ATP_free_/ADP_free_ adenylate translocation potential; and MgATP/AMP_free_ allosteric regulation driving potential. Numerous enzymes are regulated by the MgATP/MgADP ratio, while adenylates are translocated via membranes as free species, and free AMP and/or free ADP operate in cell metabolism as allosteric effectors [17,55]. The set of free and Mg-bound adenylates plus free magnesium, reflecting the real energy charge of the cell, is established in cell compartments depending on the metabolic fluxes of synthesis and utilization of adenosine phosphates within the pool of total adenylates and magnesium.

Transporters for Mg^2+^ and adenylates in all kinds of membranes (Figure 3 and Figure 4) are most likely involved in Mg^2+^-signaling simply by regulating the intracellular concentrations of Mg-free and Mg-chelated adenylates, which are equilibrated by AK. The Mg^2+^ transporters are perhaps even more important, because there must be an upper limit to [Mg^2+^] in a given metabolically active cellular compartment to prevent Mg^2+^ toxic effects. On the other hand, excess of Mg^2+^ may have some beneficial effects under specific stress conditions, e.g., in alleviating the sensitivity of plants to salinity (see below) [117]. Additionally, because adenylates are transported as Mg-free species [100], the [Mg^2+^] on both sides of a given membrane must have a crucial effect on the rates of the translocation.

It has been proposed that differences in intracellular [Mg^2+^] between cytosol and mitochondria are the key factor in the regulation of cell respiration [63,67]. It was shown that, in heterotrophic sycamore (*Acer pseudoplatanus* L.) cells, ADP is less complexed with Mg^2+^ in the cytosol than in mitochondrial matrix due to a low [Mg^2+^] in the cytosol, while ATP is mostly complexed by Mg^2+^ in both compartments. Depletion of Mg^2+^ (after growth on Mg-free media) increases free ADP concentration in the cytosol and matrix, leading to a decrease in coupled respiration and a suppression of cell growth. The [Mg^2+^] established under the control of AK mediates the ADP/ATP exchange between the cytosol and matrix, MgADP-dependent mitochondrial ATP synthase activity, and cytosolic free ADP homeostasis [67].

Marked changes in [Mg^2+^] also accompany the so-called induction phase of photosynthesis, reflecting an early response of the photosynthetic apparatus to dark-to-light transitions [55,128]. The buildup of ATP upon illumination causes the depletion of Mg^2+^ to very low values, initially equilibrated by AK to the level of ~0.2 mM in chloroplasts and cytosol. Then, in the course of transition to steady-state photosynthesis, chloroplastic [Mg^2+^] increases to 1–3 mM upon the involvement of mitochondria in the reoxidation of photosynthetically formed redox equivalents via the malate valve [55]. The estimation of intracellular [Mg^2+^] during photosynthetic induction and steady-state photosynthesis was possible because of the data on total adenylate contents obtained by rapid fractionation of protoplasts [129,130,131]. The phenomenon of photosynthetic induction, which is characterized by the delay of photosynthesis upon illumination, can be also partly explained by the depletion of Mg^2+^, affecting the activity of essential photosynthetic and respiratory enzymes.

AK controls the concentration of AMP, which serves as a cofactor of the mammalian and yeast AMP-activated protein kinase (AMPK), which in turn plays a central role in the regulation of energy metabolism [132]. Plants contain SnRK1 protein, which is an ortholog of AMPK [133]. Although free AMP allosterically activates AMPK, Mg^2+^ may participate in the catalytic mechanism of this enzyme as an indispensable cofactor [134,135]. The energy status of a cell regulates AMPK activity in a complex way, which involves tighter binding of AMP_free_ than of ADP_free_ and of Mg-bound nucleotides [136], while Mg^2+^ likely exerts a regulatory role on the enzyme. Even at high [Mg^2+^], most AMP exists in a free form (Figure 1A), and thus AMPK can be efficiently activated by AMP upon wide ranges of [Mg^2+^]. The interplay between AMP release, free Mg, and ATP production needs further investigation, in particular for plant SnRK1.

### 9.4. [Mg^2+^] under Anoxia

Under normal conditions, ATP (and other nucleoside triphosphates) is tightly bound to magnesium, thus contributing to a relatively low [Mg^2+^] status. Under stress conditions, however, there is frequently an increase in [Mg^2+^] and other divalent cations, including Ca^2+^, reflecting lower levels of ATP produced in stressed tissues. This happens especially during anoxia (lack of oxygen), when mitochondrial oxidative phosphorylation is not working, and most energy can be acquired only via glycolysis. Under these conditions, the increased [Mg^2+^] leads to the activation of Mg^2+^-requiring enzymes and redirects the energy metabolism from ATP to PP_i_-utilization [50].

The decrease in ATP production and the subsequent release of Mg^2+^ under anoxia make PP_i_ an efficient alternative energy currency. Mg^2+^ can bind to PP_i_ in two ways—as MgPP_i_ and Mg_2_PP_i_ (Figure 1B)—and it does so at a higher concentration than with ATP. The ratio between PP_i_, MgPP_i_ and Mg_2_PP_i_ is under pH control [15]. Proton pumping vacuolar PPase uses Mg_2_PP_i_ as a substrate and thus becomes active under oxygen deficiency [112,113]. Other enzymes active under anoxia use Mg-complexed substrates which bind magnesium weakly, such as phosphoenolpyruvate (PEP) or isocitrate [15]—thus, the [Mg^2+^] parameter is critical for their operation. The importance of PEP turnover under anoxia is determined by the availability of Mg^2+^, and this metabolite is directly involved in the production of PP_i_. Under anoxia, the formation of PP_i_ by “coupled” reactions of pyruvate phosphate dikinase and pyruvate kinase supports glycolysis under conditions of low [ATP] [50,137]. Another important protein, nitrate reductase, is upregulated under oxygen deficiency, whose activity is controlled by phosphorylation, in a process mediated by Mg^2+^ and 14-3-3 proteins [138].

### 9.5. Magnesium versus Calcium, Sodium and Aluminum

Changes in [Mg^2+^] as a feedback of the equilibrium governed by AK (and perhaps also NDPK and other nucleotide kinases) result in corresponding changes in internal [Ca^2+^] due to the chelation of Ca^2+^ with nucleotides to nearly the same extent as with Mg^2+^. Magnesium allosterically activates Ca^2+^ binding to calmodulin, with the latter regulating target proteins in response to sub-micromolar changes in [Ca^2+^] [139]. In turn, changes in [Ca^2+^] in a given compartment can modulate internal [Mg^2+^], in a millimolar range [42,140]. Ca^2+^ is chelated by adenine nucleotides to nearly the same extent as Mg^2+^ [141]; therefore, the intracellular [Ca^2+^] is controlled by the AK equilibrium and that of other nucleoside kinases [142]. This keeps the ratio of [Ca^2+^]/[Ca_total_] at the same level as [Mg^2+^]/[Mg_total_] despite the fact that the total concentration of Ca^2+^ is a few orders of magnitude lower than that of magnesium [142,143]. The release of Mg^2+^ when ATP level drops corresponds to an increase in internal Ca^2+^ [144].

In the IMS of mitochondria, the increase in [Ca^2+^] that accompanies Mg^2+^ release subsequently leads to the activation of multiple Ca^2+^-regulated enzymes. These enzymes include the external NADPH and NADH dehydrogenases of mitochondria, internal NADPH dehydrogenase of mitochondria [145], NAD kinase of the IMS of mitochondria [146], glutamate decarboxylase, cysteine proteases (calpain), Ca/phospholipid-dependent protein kinases, etc. [140]. Mg^2+^ counteracts with Ca^2+^ in the regulation of guard cell opening [147].

High [Mg^2+^] is known to ease saline (NaCl) stress [148,149]. Interestingly, salinity (NaCl) stress was reported to affect, in a tissue-dependent manner, the ratio of AK/NDPK [150]. This suggested that, under Na^+^ excess, different tissues fine-tune their levels of nucleotides to cope with new metabolic requirements. It is possible that internal Mg^2+^ may be involved in these rearrangements, because [Na^+^] is known to affect [Mg^2+^], and vice versa [117]. Na^+^ has been reported to displace binding of Mg^2+^ to several enzymes which specifically require Mg for activity [148].

In rice, sorghum, and several other species, even a relatively low cytosolic [Mg^2+^] can ameliorate toxic effects of aluminum ions (Al^3+^). For instance, the activity of the plasma membrane MGT1 transporter in rice increases upon Al addition to the roots, to prevent the Al-dependent inhibition of root elongation [151]. Both Al^3+^ and Mg^2+^ ions are believed to compete in binding to various cellular components, including the cell wall and plasma membrane [151]. Mg-dependent processes have also been implicated in an increase in organic acids, e.g., citrate, which is involved in alleviating Al toxicity by exudation, or in controlling cytosolic pH via regulating H^+^–ATPase activity [2,68].

### 9.6. Summary of Mg^2+^ Signaling in Plants

**Figure 5:**
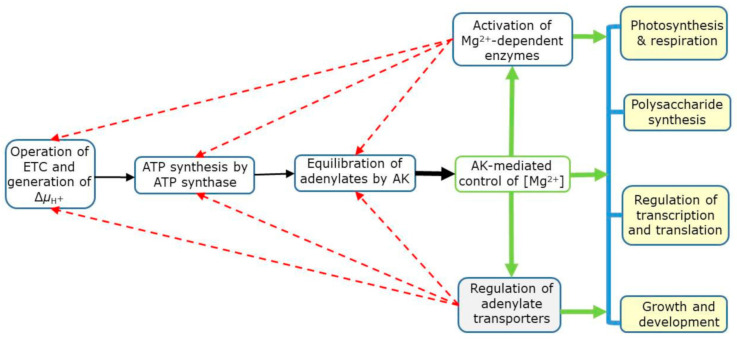
A simplified view of the Mg^2+^ role as enzyme substrate/cofactor and as a signal arising from adenylate pools. Abbreviations: AK, adenylate kinase; Δ*μ*_H_+, membrane electrochemical potential; ETC, electron transport chain. Red dotted arrows refer to feedback control by Mg^2+^.

As outlined in Figure 5, ATP is synthesized via the oxidative and photosynthetic phosphorylation in mitochondria and chloroplasts, respectively, which is a consequence of electron transport activity and the generation of membrane potential (Δ*µ*_H_+) [12,17,62]. The AK then equilibrates adenylates and establishes [Mg^2+^] in cell compartments. Mg^2+^, in turn, regulates Mg-dependent enzymes and controls activities of adenylate transporters, which use free adenylates [100]. The Mg-dependent enzymes have a direct impact on various biochemical reactions and physiological processes, including the regulation of transcription and translation [125], photosynthesis and respiration [12,17,50,51,55,62], polysaccharide synthesis [42], and eventually affecting overall growth and development (see ref 49 and references therein). The operation of electron transport chain (ETC), ATP synthases and AK is itself under the feedback control of free Mg^2+^ concentration (shown by dotted arrows in Figure 5).

## 10. Does AK Control Mg^2+^ Signaling in Other Organisms?

Although in this review we focused on plants, we are confident that Mg^2+^ signaling resulting from AK equilibrium and equilibria of related nucleotide-metabolizing enzymes is operating in all types of organisms. AK is an ancient enzyme which is widespread in all three kingdoms of life—archaea, bacteria, and eukarya [152]—and we are not aware of any group of organisms lacking AK activity. Even though the first determination of adenylate-related changes in internal [Mg^2+^], based on AK *K*_app_, was done for blood erythrocytes [53], those studies were not followed up by experimental nor theoretical research focused on AK-control of cellular [Mg^2+^] in animals. While it is unknown whether AKs in other organisms have as prominent a role in Mg^2+^ signaling as in plant cells, those AKs, by definition, are certainly involved in adenylate equilibrium and, perhaps, in equilibrating other nucleotides. The latter property was shown, for example, for *Escherichia coli* AK, which was found to have a bifunctional role as both AK and NDPK [43]. AKs equilibrate the nucleotide pools, therefore the resulting changes of [Mg^2+^] can be regarded as an unavoidable consequence of this equilibrium.

More studies, both experimental and theoretical, are required to assess the AK-controlled Mg^2+^ signaling in different organisms, both eukaryotic and prokaryotic. This should also take into account organ- or organism-specific types of metabolism. For instance, the essential differences in plant and animal metabolism are partially grounded in the aspects of nucleotide equilibria, which in many animal cell types are under the buffering control of creatine kinase reaction, which is enzymatically “coupled” with that of AK., e.g., as in a muscle. In the process, creatine kinase converts ADP back to ATP, assuring fast and active energy conversion. This coupling in animal cells pushes ATP/ADP ratios to high values, affects adenylate translocation and keeps Mg^2+^ at low levels [153]. The equilibrium constant of creatine kinase, considering its dependence on pH and other parameters, enables the calculation of free and Mg-bound adenylates, and performing the quantification of organ and tissue bioenergetics [154].

## 11. Conclusions

The most obvious and basic case of magnesium signaling is the increase in [Mg^2+^] upon the decrease in energy charge [55]. It results in the increase in [Mg^2+^] from the sub-millimolar to millimolar values in cell compartments, which leads to the regulation of many Mg-dependent enzymes and affects the operation of adenylate transporters. The release of Mg^2+^ occurs via the action of AK and triggers many processes that are regulated by [Mg^2+^], including the rate of photosynthesis, respiration, polysaccharide synthesis, stomatal opening, etc. The concentrations of other essential cations, such as Ca^2+^, Mn^2+^ or K^+^ [56], are also dependent on AK equilibrium as well as on equilibria of some other enzymes using nucleotides as substrates. The whole cellular metallome [155] depends on the balance of free and cation-bound nucleotides. Binding constants of metals with adenylates and other compounds depend on changes of [H^+^], which is a feedback signal of the equilibrium of pyridine nucleotides [14,156]. The interplay between redox and energy transformations triggers many signaling events (e.g., Mg^2+^-, sugar- and Ca^2+^- signaling, among others) [140,157,158] that initiate and regulate growth and development. Nucleotides represent the core of this signaling system, and their equilibria determine its stable and predictable operation that can be computed in the plant metabolomics framework [17]. The role of thermodynamic buffering (and adenylate equilibrium in particular) is also becoming evident in the evolutionary context [49,159].

The unique role of AK is related to its fast equilibration of adenylates and, as it is now apparent, affecting [Mg^2+^] as a feedback signal. By means of this equilibration, AK monitors and integrates different signals to ensure energy homeostasis in response to a broad range of challenges. It acts as a powerful thermodynamic buffer enzyme [48] that optimizes energy metabolism and maintains the stable and continuous operation of MgATP synthesis and consumption. It is a unique hub regulating [AMP], which itself serves as allosteric effector of essential reactions of cellular metabolism [20,21]. It appears now that AK is also at the heart of Mg^2+^ signaling. The protein that at one time was considered as just a housekeeping enzyme has indeed come a long way.

## Figures and Tables

**Table 1:** Subcellular location and roles of Arabidopsis adenylate kinases (AKs). Classification of AK1-8 generally follows that of Lange et al. [25], with the exception of nuclear isozyme AK6, which replaced what is presented here as AK8. Mitochondrial location most likely refers to the presence of AK in mitochondrial intermembrane space (IMS), but not the matrix. C, cytosol; M, mitochondria; N, nucleus; P, plastids.

AK Name and Gene	Location	Function	Reference
AK1 (*At2g37250*)	M ^(a)^, P ^(b)^	control of growth	[24,25,30]
AK2 (*At5g47840*)	P	plastid development	[24,25]
AK3 (*At5g50370*)	M ^(c)^, C ^(d)^	unknown	[25,28]
AK4 (*At5g63400*)	M	unknown	[28]
AK5 (*At5g35170*)	P	no phenotype for the knockout	[25]
AK6 (*At5g60340*)	N	control of stem growth;	[26]
		control of root growth; ribosome maturation	[31]
AK7 (*At3g01820*)	M	unknown	[25]
AK8 ^(e)^ (*At2g39270*)	M	unknown	[25]

^(a)^ [25,30]; ^(b)^ [24]; ^(c)^ [28]; ^(d)^ [25]; ^(e)^ referred to as AMK6 in ref [25].

**Table 2:** [Mg^2+^] in cellular compartments and methods used to measure [Mg^2+^].

Compartment	[Mg^2+^], mM	Method	Reference
Cytosol	0.25	^31^P-NMR	[67]
	0.40	^31^P-NMR	[64]
	0.9	Ionophore	[68]
	0.2–0.4	From *K*_app_ of AK	[55,62]
			
Mitochondria	2.4	^31^P-NMR	[67]
	1.0–3.0	From *K*_app_ of AK	[55]
			
Chloroplasts	0.5–2.0	Ionophore	[66]
	1.0–3.0	Ionophore	[65]
	0.2–5.0	From *K*_app_ of AK	[55]
			
Vacuole	5–80 ^(a)^	X-ray analysis	[2,69]
ER lumen	Unknown		
Peroxisomes	Unknown		

^(a)^ The value of 80 mM was obtained by feeding leaves with high Mg–sap solutions.

## Data Availability

Not applicable.

## Abbreviations

AEC	Adenylate energy charge
AK	Adenylate kinase
AMPK	AMP-activated protein kinase
CBL	Calcineurin B-like protein
CIPK	CBL-interacting protein kinase
ER	Endoplasmic reticulum
IMS	Intermembrane space
*K*_app_	Apparent equilibrium constant
*K*_true_	True equilibrium constant
MGT	Magnesium translocator
NDP	Nucleoside diphosphate
NMP	Nucleoside monophosphate
NTP	Nucleoside triphosphate
NDPK	Nucleoside diphosphate kinase
P_i_	Inorganic phosphate
PPase	Pyrophosphatase
PP_i_	Inorganic pyrophosphate
SuSy	Sucrose synthase
UGPase	UDP-glucose pyrophosphorylase
